# The Role of Nanomedicine in Cell Based Therapeutics in Cancer and Inflammation

**Published:** 2012

**Authors:** Rasika M. Samarasinghe, Rupinder K. Kanwar, Jagat R. Kanwar

**Affiliations:** Nanomedicine-Laboratory of Immunology and Molecular Biomedical Research (NLIMBR), School of Medicine, Faculty of Health, Deakin University, Geelong, Australia.

**Keywords:** Nanomedicine, cell therapy, cancer, blood brain barrier, inflammation

## Abstract

Cell based therapeutics is one of the most rapidly advancing medical fields, bringing together a range of fields including transplantation, tissue engineering and regeneration, biomaterials and stem cell biology. However, traditional cell-based therapeutics have many limitations, one of which is their harmful effects exhibited on healthy body cells due to their lack of specificity. Nanomedicine is providing an alternative treatment strategy that is more targeted and specific to a range of diseases. Varying from polymers conjugated with drugs or tissue targeting molecules, to proteins encapsulated within a polymer shell, nanomedicine will without a doubt play a major role in designing effective cell-based therapeutics that can overcome certain classical problems. These may include from addressing the problem of non-specificity of contemporary treatments to overcoming mechanical barriers, such as crossing cell membranes. This review summarises the recent work on nano-based cell therapy as a regenerative agent and as a therapeutic for cancer and neurological diseases.

Traditionally cell-based therapeutics were mainly considered to be through blood transfusions and bone marrow transplants, but now major advances such as genetically engineered cells, using cells for drug delivery, cancer therapies, stem cell therapy, tissue engineering and cell replacement therapy are coming into the picture (1). Nanomedicine is a developing field that uses nanotechnologies in medical applications, with highly specific medical intervention at the molecular level to cure diseases or repair tissues. Nanomedicine can be used for targeted drug delivery, imaging and diagnosis, to identify, replace and repair cells and to develop therapeutics that can minimize the systemic effects that are otherwise caused by conventional treatments such as chemotherapy (1-2). The use of nanodrugs when treating disease will create a dose differentiation between the targeted site and the rest of the body, maximising the therapeutic effects in the target area, while minimizing side effects on the rest of the body (3). The physical parameters of nanoparticles can affect their uptake by cells, with the potential to induce cellular responses. The binding and activation of membrane receptors and the subsequent internalization of particles is dependent on nanoparticle size (Fig.1) (4).

When designing nanoparticles for medical applications, potential problems such as long-term toxicity, the ability to eliminate the nanoparticles from the body within a reasonable timescale, and avoidance of non-specific background fluorescence when monitoring the nanoparticles *in vivo* need to be taken into consideration (5). As more research is done into the use of nanoparticles in clinical applications, these potential issues are being addressed.

Traditional methods for cell or tissue transplants have been limited and at times unsuccessful due to immune attacks activated by the host defence system, hence nanotechnologies are in progress on developing strategies that can avoid these situations. Two main approaches used by nanoparticles involve encapsulating drug molecules or cellular components within self aggregating monomers such as liposomes, micelles and polymeric nanoparticles, that allows protection of molecules from serum and gradual release of drugs. Another method uses the approach of covalently tagging nanoparticles with drug molecules and targeting moieties using cleavable linkers that can enhance specificity of the nanoparticles to targeted sites and increase drug effectiveness (3).

## Nanoparticles to prevent immune activation

Nanosized porous materials can be used for the immunoisolation of transplanted cells. Encapsulation of cells within a semi-permeable membrane allows the transport of oxygen and flow of nutrients between the host environment and the introduced cells, but blocks larger immune cells such as leukocytes and monocytes from attacking the transplanted cells (6).

Molecular camouflage is another method used, which attaches polymer chains, usually PEG (polyethylene glycol) chains, to the surface of the cell or tissue, which prevents cellular recognition by creating a barrier with the chains. The attachment of the polymer chains can occur by coupling the polymer to amines of proteins or carbohydrates on the cell surface, or by directly inserting a polymer-lipid conjugate into the membrane (1, 7).

PEGylation of nanoparticles allows them to escape the reticulo-endothelial system, which is a significant biological barrier (8).

**Fig 1:**
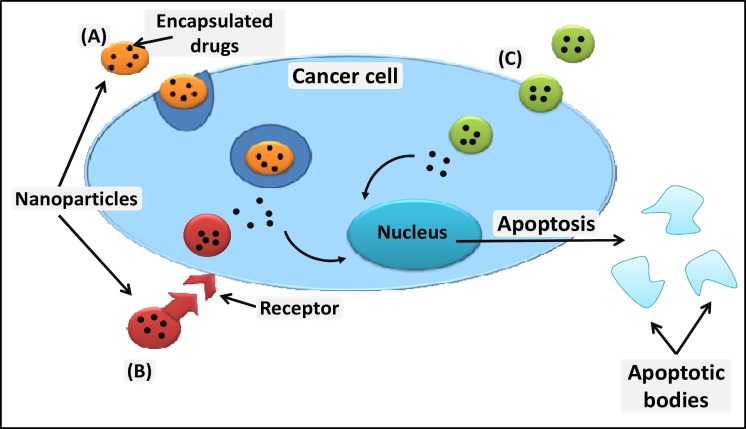
Nanoparticles can enter a cell either by simply crossing the plasma membrane, by endocytosis (A), receptor mediated endocytosis (B) or through absorptive pinocytosis mechanism (C), where a specific ligands are attached to nanoparticles enabling them to target cells with a specific receptor

The addition of PEG molecules forms a hydrophilic layer on the surface of the nanoparticles, creating a steric stability, preventing aggregation of nanoparticles with each other and with components of the blood (8), allowing better biodistribution. Electrostatic layer-by-layer assembly of an ultra-thin film has been used to coat cells and prevent host immune attack. The cell membrane, which is negatively charged, is coated with a cationic polymer shell – the opposite charges attract (1). The composition and thickness of the cell coating can be varied for different applications. Poly(lactide-co-glycolide) (PLGA) nanoparticles are being studied as a potential drug delivery system, and have shown very promising results. PLGA shows no cytotoxicity, as it undergoes hydrolysis in the body, forming biocompatible fragments which are then metabolized and removed from the body through the citric acid cycle (9).

PLGA nanoparticles are also able to cross the Blood Brain Barrier, and so show promise in treating neurological and psychiatric disorders, as well as other diseases (9). Nanocrystals are crystalline form of a drug, formed from aggregation of the drug molecules, surrounded by a thin coat of a surfactant (10). The nature of the coating on the nanocrystals determines the biological reaction, but a hydrophilic coating increases bioavailability and biodistribution, and prevents the crystals aggregating (10). Therefore, high doses of a drug can be achieved using nanocrystals, and by coating with nanomaterials poorly soluble drugs can have a higher bioavailability (10).

Therapeutic proteins have also shown potential for the treatment of cancers and other diseases, however the application has been limited due to poor stability and low permeability of the protein to cell. A nanoparticle with a protein core and a thin, permeable polymer shell has been developed to overcome these limitations. It can either be degraded by the body or remain stable, depending on the purpose (11). The degradable nanoparticles can be used to deliver proteins into a targeted cell, so the protein can be released once inside its target. Non-degradable nanoparticles which show long term stability without toxicity to the body (11).

Glyconanoparticles are new nanotools at the forefront of biomedical research. Carbohydrates are used in a functional, rather than protective role, encoding biological information. Glyconano-particles have been used to study carbohydrate-mediated interactions, and used in cellular labelling and targeted imaging, as magnetic and fluorescent probes. Glyconanoparticles are favourable for a number of reasons, including the fact that glycocalyx covers eukaryotic cells, and controls some cellular communication, cells will uptake ‘glyco’ easily and favourably as they need sugars to survive, and both simple and complex carbohydrate molecules are soluble in water. Carbohydrates can also minimize nonspecific adsorption and immunogenic responses (12).

Inorganic nanoparticles are usually composed of silica or alumina, with a range of possible cores, including metals, metal oxides and metal sulphides (10).

They can be made to avoid the reticuloendothelial system, by varying the size and/or surface composition of the nanoparticles (10). While inorganic nanoparticles do not have the flexibility of liposomes and polymeric nanoparticles, they have a range of useful functions. Iron oxide nanoparticles are very effective for magnetic resonance imaging, and have been used to trace a range of transplanted cells, including bone marrow cells, neural cells and embryonic stem cells (2). Superparamagnetic iron oxide nanoparticles (SPION) have been loaded with Doxorubicin in an attempt to overcome the traditional limitations of doxorubicin, that is, strong side effects and cell resistance. While the SPIONS alone did not show any cytotoxicity, the DOX-SPION induced high rates of cell death, higher than DOX alone at a higher dose (13).

After cells have been transplanted it is necessary to be able to image them to monitor their survival, function and location. Fluorescent nanoparticles are an effective means of this imaging, as are quantum dots (QDs). Quantum dots are nanosized crystals that can display a range of colours. This means a range of proteins, or DNA sequences can be tagged with different coloured quantum dots to view a number of particles in one image (2).

## Nanotechnology and Regenerative Medicine

Regenerative medicine focuses on restoring cells and extracellular matrices that have been lost or damaged, or are no longer functional. It is an important field of medical research, as the ability to regenerate cells in the central nervous system has the potential to reverse paralysis due to spinal cord injuries, and to help those suffering from stroke, Alzheimer’s disease, Parkinson’s disease and Multiple Sclerosis (14), to name a few.

 Regenerative medicine can also help heart disease and heart failure, as well as developing new vasculature. Regeneration of pancreatic β cells could help diabetics, eliminating their need for insulin injections (14). The potential of regenerative medicine to help people is almost endless, including regeneration of teeth, as well as musculoskeletal elements, including bone, tendon, ligaments and cartilage (14).

Nanosized titanium implant surfaces have helped promote the response of bone cells, leading to faster calcium deposition, which improves the integration with surrounding bone, promoting faster and more effective regeneration (15). PLGA has been shown to promote chondrocyte adhesion and proliferation in cartilage for faster regeneration (15). Also the porous property of chitosan/collagen scaffolds loaded with transforming growth factor β1 was shown to promote cartilage regeneration in rabbits by activating chondrocyte growth and matrix synthesis (16).

Much work is also being done on cardiac nanomedicine. Zhong et al. (2005) created nanosized structured scaffolds from PLLA and PLGA nanofibres. Cardiac myocytes adhered to these scaffolds, and aligned with the fibre orientation. The cells exhibited a preference of PLLA over PLGA, and within seven days after cells were introduced to a PLLA scaffold they responded to external stimuli as a cohesive electrical unit (17).

## Nanomedicine and cancer

Cancer is one of the primary targets for nanomedical research. Because ‘cancer’ is so broad and every clinical representation is different there is a need to develop individualised diagnoses and treatments. The current therapeutic agents for cancer have an inappropriate biodistribution, and suffer from poor pharmacokinetics. Most anticancer drugs have a low molecular weight, and due to this are often rapidly cleared from the circulation before reaching the target site. They do not accumulate well in tumours and due to this often have a toxic effect on many healthy tissues in the body (18).

A strategy used in cancer treatments is identifying a patient’s disease biomarkers and customizing targeted treatments according to the expressed biomarkers. A cancer biomarker could be a mutant gene, a protein, lipid, RNA, carbohydrate or small metabolite molecule. After these biomarkers have been identified, multifunctional nanoparticles will be able to detect and image tumours, as well as treat them and then be used to monitor progression after treatment (8).

Nanoparticles often accumulate in tumours through passive targeting, due to the enhanced permeation and retention (EPR) effect. This effect is due to leaky vessels in tumours caused by the fast and ineffective angiogenesis during tumour growth and the lessened lymphatic drainage, which decreases the re-uptake of molecules into the bloodstream (19). These factors allow the retention of nanoparticles within the tumour long enough for disintegration of the nanoparticle to release its contents (8). 

The first nanoparticles used for cancer therapy were liposomes. They consist of a lipid bilayer surrounding an aqueous core and encapsulating the drug (8). Liposomes are biocompatible, biodegradable, and can carry both hydrophobic and hydrophilic molecules (2). Their main advantages are their ability to pass easily through cell membranes, to bind to targeted sequences, and to easily bind with DNA or RNA for transfection (2).

Many liposomal targeting strategies have been developed to target endothelial cells, due to their accessibility and their critical role in angiogenesis (20). The most effective way to target liposomes to neovasculature is the inclusion of positively charged lipids. Cationic complexes, including lipids in a complex with DNA, have been shown to aggregate intravascularly. The particles are cleared mainly by macrophages in the liver and spleen, but also by certain endothelial cells (20). This was an important discovery for the design of target lipid nanoparticles. A study by Eichhorn et al. (2004), showed that if positively charged protamine is administered before cationic liposomes were injected, the binding of liposomes to tumour vasculature was doubled, but binding to quiescent endothelium remained low (21). Studies on mice with pancreatic islet cell carcinoma showed that cationic liposomes were taken up by activated angiogenic endothelial cells at a degree 15-30 times higher than that of quiescent endothelial cells (22).

These studies show strong promise for the use of positively charged liposome nanoparticles in tumour suppression, by targeting the tumour’s blood supply with minimal effect on healthy endothelial cells. Another example of this was doxorubicin, a chemotherapeutic drug, which also causes cardiotoxicity, limiting the dosage that can be administered. Doxorubicin was encapsulated within anionic liposomes, which increased the accumulation of the drug-loaded nanoparticles within tumours, and reduced cardiotoxicity. A new lipid-based nanoparticle that is being developed is the solid lipid nanoparticle (SLN). SLNs are efficient and non-toxic, and can carry either lipophilic or hydrophilic drugs, while effectively controlling the release of these drugs (8).

Dendrimers are the smallest form of organic nanoparticles. They are branched polymeric molecules with a three dimensional geometric pattern (8). Dendrimers form electrostatic interactions with the negatively charged phosphate backbone of DNA (2). This means the DNA is packaged for efficient delivery to cells (2). Dendrimers are used for a range of diagnostics and therapeutics in cancer, as well as for photodynamic therapy and hyperthermia therapies using AuNPs. They can carry drugs either by encapsulation or conjugation, but have a major limitation in drug release. Encapsulated drugs are often released quickly, before reaching the target site, and conjugated drugs are released according to their chemical bond to the dendrimer (8). 

Even with this limitation dendrimers have many advantageous properties, including their surface functionality, which allows selective binding of agents for imaging, targeting or other components to increase target specificity (8).

Superparamagnetic iron oxide nanoparticles have been used to improve the accuracy of cancer staging, better define primary tumours, view angiogenesis and detect metasases (19). They can also be guided to target sites using external magnetic fields (8). They can passively accumulate at tumour sites, again due to the EPR effect. At the tumour site an external near infra-red laser can be used to heat the metal nanoparticles, destroying the tumour tissue while leaving surrounding normal tissue healthy. From this, gold nanorods have also been developed, which are shown to release heat faster and more effectively than spherical metal nanoparticles (8). 

The role of angiogenesis has also been a focus of recent research in B-Chronic Lymphocytic Leukemia (CLL). It has been shown that a VEGF signalling pathway is the site of apoptosis resistance in CLL B cells. It was found that there was significantly more apoptosis in CLL B cells when cultured with an anti-VEGF antibody. To improve the efficiency of these antibodies, they have been conjugated with gold nanoparticles. Gold nanoparticles were chosen because of their high surface area, surface functionalisation, and ease of characterization (23).

The gold nanoparticle-VEGF-antibody conjugate showed a significant down-regulation of anti-apoptotic proteins. Cells treated with the nanoparticle-antibody conjugate showed internalization of the nanoparticles in endosomes and in multivesicular bodies. Studies showed a significant increase in apoptosis when treated with the nanoconjugates, compared to just the gold nanoparticles or just the antibody complex (23).

Research is also being undertaken to use gold nanoparticles to enhance the radiofrequency thermal destruction of human gastrointestinal cancer cells non-invasively. Radiofrequency ablation (RFA) is currently used to treat some malignant tumours, however it is an invasive procedure with only a ~60% success rate. The treatment is also non-specific, with normal tissues also being damaged in many cases (24).

Gold nanoparticles were selected to develop a non-invasive, more targeted radiofrequency therapy. Gold nanoparticles were chosen because of the above mentioned properties, as well as their anti-angiogenic properties. It was proposed in the study that gold nanoparticles would release significant amounts of heat when exposed to a radiofrequency field. Gannon et al. (2008) performed an *in vitro* study that showed the success of gold nanoparticles in this application. They were taken up by cells in culture, and localized in vesicles within the cytoplasm. The gold nanoparticles did not produce any cytotoxic effects and did not seem to affect proliferation of the cells. The cells with gold nanoparticles were exposed to an external radiofrequency source, and the nanoparticles released significant amounts of heat. This exposure produced a dose-dependent lethal injury in >96% of the targeted cells (24). These results show promise for *in vivo* studies and potential clinical applications in the treatment of a range of malignant tumours. Table 1 shows the various anti-cancer drugs and the nanoformulations used to target different cancers. 

## Cancer Stem Cells

Another landmark on the road to curing cancer has been the discovery of cancer stem cells (CSCs). Cancer stem cells have similar properties to normal stem cells in that they are able to give rise to all cell types, but in the case of cancer stem cells, only all cell types are found within the tumour. They are therefore tumorigenic cells. They generate tumours through the stem cell processes of self-renewal and differentiation to multiple cell types. Cancer stem cells have become a major research focus due to their ability to form new tumours and relapse in patients that have had tumours removed. There have been two main strategies developed for CSC cancer therapies. One is the exposure of CSCs to high levels of conventional cytotoxic agents. The other is developing new therapies that target the cancer stem cells. The use of conventional cytotoxic agents is limited due to their lack of target specificity.

The effect of increasing the dose for CSCs will also increase the dose for the rest of the body, making it dangerous. Therefore, increased exposure needs improved specificity, either by using nanoparticles, or by overcoming transport resistance mechanisms (3). One of the main problems with drug transport has been transporter pumps that pump substrate drugs out of the cell, thereby decreasing the effective drug concentration within the cell. ABC transport systems are one of the main hindrances related to drug delivery. Most small molecules drugs enter the cell by diffusion, and hence are affected by these transport systems. Nanodrugs enter the cell via endocytotic vesicles, avoiding the drug transport systems on the cell membrane (3).

**Table 1:** Nano-forms of anti-cancer drugs

Drug	Nano-drug	Target/action	Reference
Daunomycin	DaunoXome (liposome)	Leukaemia	(25)
Doxorubicin	Doxil/ Caelyx-encapsulation in PEGylated liposome Myocet-non-PEGylated liposome	Acute leukaemia, lymphoma, breast carcinoma, osteosarcoma, haematological malignancies, Kaposi’s sarcoma Myocet-metastatic breast cancer in combination with cyclophosphamide	(26)
Annamycin	Liposome	Leukaemia, reticulosarcoma	(26)
Tretinoin	Loaded nanocapsules; liposomes	Acute promyelitic leukaemia	(27)
Vincristine	Liposome	Chemotherapy-nephroblastoma, lymphoma, lymphoblastic leukaemia	(28)
Cisplatin	DMPG-complexed, entrapped in liposomes	Melanomas	(26)
Hydroxyrubicin (lipophilic prodrug)	Liposome	Leukaemia, reticulosarcoma	(26)
Mitoxantrone	Cytostatic complex with lipophilic acid	Metastatic breast cancer, acute myeloid leukaemia, non-Hodgkin’s lymphoma, acute lymphoblastic leukaemia	(26)
Paclitaxel/ Taxol	Lipophilic prodrug paclitaxel oleate in sterically stabilized	Lung, ovarian, and breast cancer	(29)

The nanodrugs can then also be engineered to target cancer cells or tumours, maximising the drug exposure at the diseased site. Active targeting uses the binding of a specific target ligand to a specific molecule that acts as a receptor, whereas passive targeting relies on haemodynamic changes linked to the blood supply of tumours. Targeting of CSCs within a tumour potentially allows both active, more specific targeting, and passive, slightly more generalised targeting (3).

The development of these therapies holds promise for tumour suppression, and a significant reduction in cancer relapse after the surgical removal of tumours, if CSC therapy is found to be a viable post-surgical treatment option.

## Nanomedicine and neurological diseases

The primary obstacle in the treatment of diseases affecting the (CNS) is the Blood Brain Barrier (BBB). The BBB is composed of brain endothelial cells and separates the brain from the rest of the systemic circulation. It is the major route of entry for drugs into the CNS. The main role of the BBB is to maintain homeostasis of ions for normal neuronal function, to supply the brain with nutrients and protect it from toxic agents. 

**Fig 2:**
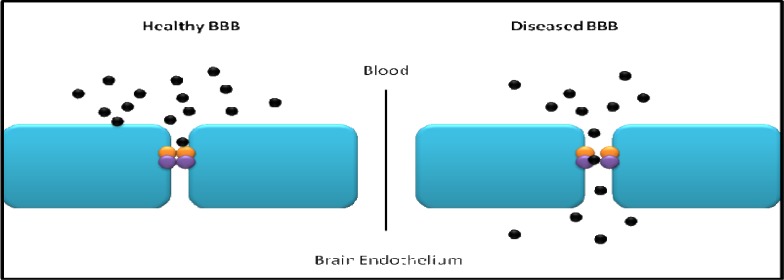
A healthy blood brain barrier has intact tight junctions to prevent unmediated passage of molecules into the brain, whereas a diseased blood brain barrier can have defective tight junctions, becoming ‘leaky’, and allowing molecules, unrestricted entry into the brain endothelium

The restriction of transport into the brain is through physical tight junction barriers, as well as through the use of enzymes as a metabolic barrier (Fig.2) (30).

**Fig 3:**
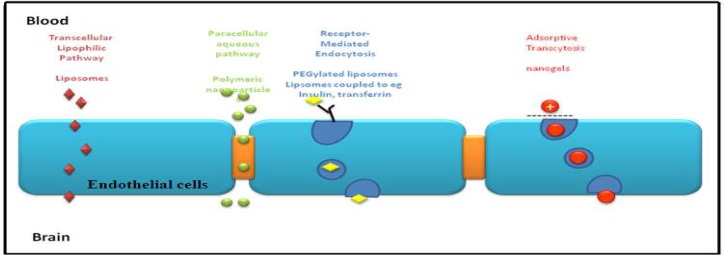
Mechanisms of transport across the BBB. Small liposomal molecules, including liposomes, can simply cross the blood brain barrier. Polymeric nanoparticles are transported through tight junctions, whereas endocytosis and transcytosis transport molecules cross the BBB in vesicles

Molecules can cross the BBB to enter the brain interstitial fluid either through lipid-mediated transport by free diffusion of micromolecules, or facilitated transport of micro and macro-molecules. The BBB and the Blood-CSF-Barrier both have efflux transport systems that can also remove substances from either the brain or the CSF and transfer them out to the systemic circulation (30). Once molecules have crossed the BBB into the cerebrum they are rapidly and extensively distributed throughout the brain, due to the high vascular density in the brain. When designing nanocarriers to cross the BBB, modifications such as recognition ligands and ligands to enhance transcytosis need to be considered (Fig.3) (31).

Modified liposomes have been used in the treatment of neurodegenerative disorders. While liposomes without modification are incapable of crossing the BBB, PEGylated liposomes conjugated to monoclonal antibodies to glial fibrillary acidic protein (GFAP) can cross the BBB at the site of disease, where it is more permeabilized (31). Liposomes can also be coupled with mannose, transferrin or insulin receptors to cross the BBB. The transferrin receptor in particular is more highly expressed in the BBB during certain pathologies, in this case after a stroke. It has been shown in a rat model that transferrin-conjugated liposomes could specifically target the post-ischemic brain endothelium after a stroke (32).

Nanoparticles coated with a PEG-containing surfactant have been used to deliver a range of drugs to the CNS, including analgesics, anti-convulsants and anti-cancer agents (31) (Table 2).

Nanoparticles conjugated with metal chelators have recently been shown to cross the BBB, chelate with metals, and exit through the BBB as a complex, which can reduce the metal toxicity in neural tissue, and potentially reduce the harmful effects of oxidative damage in Alzheimer’s and other diseases (33). Carboxylated polystyrene nanospheres where shown to remain in the vasculature after injection in normal conditions, but during cerebral ischemia-induced stress, the BBB is partially opened, and the nanospheres enter the cerebrum (34).

**Table 2:** Nano-forms of neurological drugs

**Drug**	**Nanodrug**	**Action/ Target**	**References**
Doxorubicin	PEGylated dendrimer with transferrin	G6 Glioma cells	(39)
BCNU	Magnetic nanoparticle with Fe3O4 core	Glioma	(40)
H0-1 gene	Reducible poly (oligo-d-arginine) peptide	Protect brain cells from IR related injury, including stroke	(41)
Mito-Q10	Attached to triphenyl phosphonium	Parkinson’s disease	(42)
5-chloro-7-iodo-8-hydroxyquinoline CQ	Polymeric encapsulation	Cu/Zn chelator – Alzheimer’s disease	(37)
Thioflavin-T	Butylcyanoacrylate polymer encapsulation	Alzheimer’s – detection of Aβ plaques	(37)
D-penicillamine (Cu(I) chelator)	Nanoparticle encapsulation	Reverse metal induced preceptiation	(37)

Micelles have been conjugated to antibodies, either against brain α2-glycoprotein or insulin as targeting bodies. They were both shown to deliver either a drug or a fluorescent probe to the brain, and when haloperidol was solubilised in micelles it was shown to have a much higher neuroleptic activity than as a free drug (31). It has been shown that in brain microvessel endothelial cells, insulin modified micelles undergo receptor-mediated transcytosis to cross from the blood into the brain (31). Nanogels are networks of cross-linked polymers at the nanoscale. They often combine ionic and non-ionic chains, and they network with oppositely charged molecules, such as oligonucleotides, DNA, siRNA, low molecular weight drugs, and proteins (34). Nanogels decrease the degradation of oligonucleotides in the brain microvessel endothelial cells. Nanogel surface can be modified with transferrin or insulin to improve their ability to cross the BBB (31). Alzheimer’s disease is one of the most common neurodegenerative disorders, and at this stage it has no cure. It results from the deposition of senile plaques into the synaptic spaces of the neocortex. The neurodegeneration that comes with Alzheimer’s disease affects cognition and memory, as well as behaviour. In Alzheimer’s disease atrophy of the hippocampus and cerebral cortex, and enlargement of ventricles can be seen (34). Research into nanomedicine for the treatment of Alzheimer’s and other neurodegenerative diseases is being done. Polymeric nanoparticles are being used in Alzheimer’s disease research because they can open the tight junctions, cross the BBB, they have a high drug loading capacity, and can be targeted towards the mutant proteins of both Alzheimer’s disease (34) and Parkinson’s disease (34).

Cui et al. (2005) performed an *in vitro* study of chelation of iron as a potential Alzheimer’s disease treatment. In this study D-penicillamine, a Cu(I) chelator, was conjugated to nanoparticles to reverse the precipitation of β-amyloid protein, which is induced by metals in the brain. These D-penicillamine loaded nanoparticles resolubulized the β-amyloid plaques after easily crossing the BBB due to the polymeric nanoparticles (33). This resolubilization will slow, and potentially reverse, the progression of Alzheimer’s disease, and may lead the way for similar treatments in other neurodegenerative disorders**.**

## Nanomedicine and cartilage regeneration

Joint diseases such as osteoarthritis (OA) is a combination of several disorders in which the physiochemical properties of the cartilage are altered leading to tissue softening and damage. The imbalance of anabolic and catabolic factors within the cartilage activates the production of cytokines and inflammatory mediators that gradually lead to the degradation of cartilage components. Furthermore, due to the aneural and avascular nature of the joints, delivery of drugs to the affected area becomes a more challenging process. Studies utilizing nanotechnology has shown some interesting progress in repairing and treating degraded cartilage.

Chitosan nanoparticles have been widely reported to help with cartilage synthesis and chondrocyte growth. Chitosan conjugated with hyaluronic acid to produce a hybrid polymer based matrix for chondrocytes showed increased cell adhesion, cell growth and synthesis of aggrecan and collagen as compared to the chitosan groups alone (39). In a another study, it was shown that β-chitin or pure chitosan scaffolds were able to maintain growth of chondrocytes and that within 4 weeks a cartilage like layer similar to the hyaline cartilage was formed (39).

An important aspect of assessing the success of cell transplantation within a matrix would be to evaluate its distribution and activity within the construct. Labelling of chondrocytes with superparamagnetic iron oxide nanoparticles have demonstrated the advantages of analysing cell viability, morphology, function and distribution of cells in tissue engineered constructs and also enhancing the detection of cells using conventional methods such as magnetic resonance imaging (39-40). These studies show the potential of using nanomedicine in regenerating cartilage through effective and non-invasive methods.

## Conclusion

With an aging population and an expectation to live longer, healthier and more functional lives, there is an expectation for medicine to develop rapid and effective treatments for an ever-growing range of diseases and injuries. New applications of nanodrugs are continuously being tested and developed.

While traditional cell-based therapeutics had numerous and severe limitations, including cytotoxicity to healthy cells, poor biodistribution and poor bioavailability, nanoparticles are being explored as vehicles to deliver drugs and/or therapeutic agents to target cells with high specificity and little-to-no cytotoxicity. Nanomedicine is being used to develop natural, synthetic and biomimetic scaffolds for tissue regeneration. Traditional chemotherapeutic drugs are being conjugated to or encapsulated within a range of nanoparticles for specific delivery, avoiding traditional problems such as toxicity to healthy tissue, and removing the strict dosage limitations there used to be due to the lack of specificity.

Neurodegenerative disorders are also becoming more prominent in today’s aging society, and so there is much research being undertaken to develop methods of getting drugs across the BBB into neural tissue to treat these disorders. Nanomedicine is thus developing into a seemingly limitless field to detect, diagnose and treat a huge range of diseases and disorders. 
